# Historical Perspectives in the Development of Antiviral Agents Against Poxviruses

**DOI:** 10.3390/v2061322

**Published:** 2010-06-14

**Authors:** Erik De Clercq

**Affiliations:** Rega Institute for Medical Research, Department of Microbiology and Immunology, Minderbroedersstraat 10, B-3000 Leuven, Belgium; E-Mail: erik.declercq@rega.kuleuven.be; Tel.: +32-16-337367; Fax: +32-16-337340

**Keywords:** thiosemicarbazones, interferon (inducers), idoxuridine (IDU), acyclic nucleoside phosphonates, cidofovir, CMX001 (HDP-CDV), tecovirimat (ST-246), Gleevec (imatinib)

## Abstract

The poxvirus vaccinia virus (VV) served as the model virus for which the first antivirals, the thiosemicarbazones, were identified. This dates back to 1950; and, although there is at present no single antiviral drug specifically licensed for the chemotherapy or -prophylaxis of poxvirus infections, numerous candidate compounds have been described over the past 50 years. These compounds include interferon and inducers thereof (*i.e.,* polyacrylic acid), 5-substituted 2’-deoxyuridines (*i.e.,* idoxuridine), IMP dehydrogenase inhibitors, S-adenosylhomocysteine hydrolase inhibitors, acyclic nucleoside phosphonates (such as cidofovir) and alkoxyalkyl prodrugs thereof (such as CMX001), viral egress inhibitors (such as tecovirimat), and cellular kinase inhibitors (such as imatinib).

## 1. Introduction: The thiosemicarbazones

In 1972, D.J. Bauer stated in his “Introduction to Antiviral Chemotherapy” [1] that the first true antiviral agents discovered were the thiosemicarbazones. They were introduced into medicine by Domagk *et al.* [2], as active against tubercle bacilli. As stated by Bauer [1], “the discovery of an agent effective in one branch of microbiological science is usually followed by its trial in other branches”, and so, *p*-aminobenzaldehyde thiosemicarbazone was the first antiviral agent, found in 1950 by Hamre, Bernstein and Donovick [3] to be active against vaccinia virus (VV) infection in fertile eggs and mice. This observation was confirmed by Hamre, Brownlee and Donovick in 1951 [4]. Thompson, Price and Minton [5] then showed that benzaldehyde thiosemicarbazone protected mice infected intracerebrally with VV.

**Figure 1.**
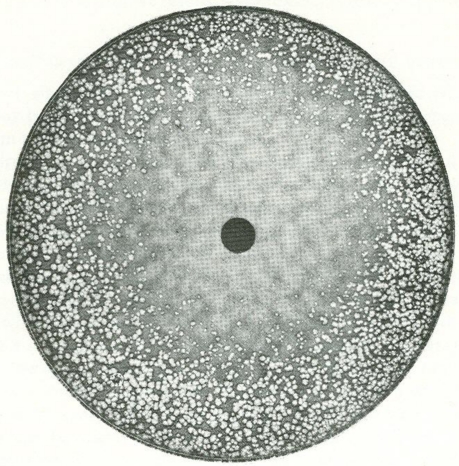
Plaque-inhibition test. The central disc contains a compound having activity against rabbitpox virus. The compound has diffused through the agar overlay and the disc is surrounded by a zone in which the formation of plaques has been inhibited. Figure taken from Bauer [1].

An example of virus plaque inhibition (in HeLa cells), a technique as originally applied in these early days [1], illustrates the central disc containing a compound having activity against rabbit poxvirus (Figure 1). The compound has diffused through the agar overlay, and the disc is surrounded by a zone where the formation of plaques is inhibited. Which compound inhibited rabbit poxvirus plaque formation is not clear from the context.

In his paper on “Thiosemicarbazones” [6], Bauer contended that the benzaldehyde thiosemicarbazones were the first true antiviral agents to be discovered and that “they could have yielded antiviral agents useful in human medicine if their development had not come to a halt”. Thompson *et al*. [7] then described a wide range of thiosemicarbazones, from which emerged isatin 3-thiosemicarbazone [8]. This compound was further studied by Bauer [9] and found to be effective against the multiplication of VV in mice infected intracerebrally. The thiosemicarbazone finally selected for use in man was 1-methylisatin 3-thiosemicarbazone, better known as methisazone (brand name: Marboran). For the structural formulae of the compounds, see the Appendix accompanying this article. As further documented by Bauer [1], methisazone had tentative efficacy in the treatment and prophylaxis of smallpox, and the treatment of the complications of smallpox vaccination, *i.e.,* eczema vaccinatum and vaccinia gangrenosa. Methisazone would have yielded better results than antivaccinial γ-globulin in the prophylaxis of smallpox in persons who had been in contact with variola virus [10]. However, with the imminent global eradication of the variola virus (resulting from the successful implementation of the smallpox vaccination), interest in the use of methisazone for both the prophylaxis or treatment of smallpox rapidly declined, and so did the interest in further developing antiviral compounds active against either variola or vaccinia virus.

## 2. The VV pox tail lesion model: the simplest virus mouse model ever described

Vaccinia virus (VV) was the first virus I worked with when joining the Laboratory of my master, Prof. P. De Somer in 1964, as a student in medicine, to work on interferon and inducers thereof. Vaccinia virus was at that time not the highest priority as a target virus for antiviral development, but it was a useful tool virus for titrating interferon, not dangerous as we were all vaccinated with the virus, and extremely stable and reliable as a challenge virus, *i.e.,* for interferon titrations in murine L929 cells, which at that time I did almost weekly. Then I found VV to be a most useful virus to demonstrate the efficacy of interferon and interferon inducers [such as polyacrylic acid (PAA)], even if the latter was administered four weeks before VV infection (Table 1) [11]. The model was simple and reliable: at 7–10 days after intravenous injection with VV, the mice developed tail lesions which could be easily counted, and the reduction in the tail lesion number could be interpreted as a quantitative measure of the antiviral activity of any given anti-VV agent. The model was first described by Boyle, Haff and Stewart [12], but I found it extremely useful, convenient and accurate to demonstrate *in vivo* anti-VV activity of interferon and interferon inducers [11], and of many nucleoside analogs as well, including ribavirin, and 5-substituted 2’-deoxyuridines [13], carbocyclic 3-deazaadenosine [14], 3’-fluoro-3’-deoxyadenosine [15], (*S*)-HPMPA [16], EICAR [17], and S2242 [18]. The VV pox tail lesion model has also been used by others [19–21] to demonstrate the anti-VV activity of a variety of compounds (as reviewed by Smee and Sidwell [22]), but, despite (and probably because of) its simplicity, the VV pox tail lesion model has since the review article of Smee and Sidwell [22] in 2003 no longer popped up in recent publications.

## 3. 5-Substituted 2′-deoxyuridines

The 5-substituted 2’-deoxyuridines idoxuridine (5-iodo-dUrd, 5-iodo-2’-deoxyuridine, IDU) and trifluridine (5-trifluoro-2’-deoxythymidine, 5’-trifluoromethyl-dUrd, TFT) have been generally viewed as initiating the dawn of antiviral therapy, now 50 years ago [23]. In essence, this is because IDU and TFT later became the first licensed antiviral agents approved for the topical treatment (as eye drops and/or ointment) of herpetic keratitis [due to herpes simplex virus (HSV)]. It should not be ignored, however, that, while Prusoff [24] was the first to describe the synthesis of IDU, Herrmann [25] and Kaufman, Nesburn and Maloney [26] were the first to describe the inhibitory effects of IDU on vaccinia virus plaque formation in chick embryo cell cultures *in vitro* [25], and vaccinia virus infection of the rabbit cornea *in vivo* [26]. In fact, IDU and TFT would only be the first of a long list of 5-substituted 2’-deoxyuridines [27,28], which also included 5-nitro-dUrd [29], 5-formyl-dUrd [30], and 5-ethyl-dUrd, 5-vinyl-dUrd and 5-ethynyl-dUrd [31], and which were all shown, in 1980, to inhibit VV replication in primary rabbit kidney cell cultures *in vitro* at an IC_50_ in the 0.1-1 μg/ml concentration range [31]. Ten years earlier, in 1969, Swierkowski and Shugar [32] had pointed to the importance of 5-ethyl-2’-deoxyuridine as an antiviral agent because it was considered nonmutagenic (in contrast with 5-iodo- and 5-bromo-2’-deoxyuridine). In 1965, Nemes and Hilleman [33] [the same Maurice Hilleman as magnificently depicted by Paul A. Offit in his book “Vaccinated. One Man’s Quest to Defeat the World’s Deadliest Diseases” [34]] described the effective treatment of herpetic keratitis with the new 5-substituted 2’-deoxyuridine 5-methylamino-2’-deoxyuridine (MADU); the antiviral activity of MADU roughly approximated that of IDU [33], but the tests were done with HSV, not VV. The largest series of 5-substituted 2’-deoxyuridines ever reviewed for their antiviral activity was that published in Mike Harnden’s book [35], but while the compounds were evaluated for their comparative activity against both HSV-1 and HSV-2, no data for VV were included [35].

Of the 5-substituted dUrd analogs, only IDU has been subject of an in-depth evaluation of its efficacy against VV infections in mice [36]. IDU had a protective effect in the VV tail lesion model (see *supra*) in immunocompetent mice, and in a lethal model for VV infection in SCID (severe combined immune deficiency) mice that had been infected with VV either intranasally, intraperitoneally or intravenously [36], but the dose at which IDU had to be given (subcutaneously) was relatively high (75–150 mg/kg/day), and when IDU was compared to cidofovir [(*S*)-HPMPC], the latter proved more efficacious at a lower dose (25 mg/kg/day) in its protective activity against intranasal VV infection in SCID mice [36].

## 4. Nucleoside analogs other than IDU and 5-substituted dUrd analogs

In addition to the 5-substituted 2’-deoxyuridines, numerous other nucleoside analogs have been reported to inhibit VV at a sufficiently low IC_50_ (<1 μg/ml) to be potentially useful from a therapeutic viewpoint [27]: *i.e.,* IMP (inosine 5’-monophosphate) dehydrogenase inhibitors, such as EICAR (5-ethynyl-1-β-D-ribofuranosylimidazole-4-carboxamide) [17]; SAH (S-adenosylhomocysteine hydrolase) inhibitors, such as neplanocin A [37], 3-deazaneplanocin A [21], 9-(*trans*-2’,*trans*-3’-dihydroxycyclopent-4’-enyl)adenine (DHCeA) and -3-deazaadenine (c^3^DHCeA) [39], (1’*R*,2’*S*,3’*R*)-9-(2’,3’-dihydroxycyclopentan-1’-yl)adenine (DHCaA) and -3-deazaadenine (c^3^DHCaA) [40], (±)-6’β-fluoroaristeromycin (F-C-Ado) [41], (±)-5’-noraristeromycin [42], (-)-5’-noraristeromycin [43], *epi*(-)-5’-noraristeromycin [44], (-)-3-deaza-5’-noraristeromycin [45], (*R*)-6’-*C*-methylneplanocin A [46], 6’-homoneplanocin A [47], and 2-fluoroneplanocin A [48]; carbocyclic cyclopentenyl cytosine (Ce-Cyd) [49,50], a putative inhibitor of CTP synthetase; 8-methyladenosine [51], whose mechanism of action as an inhibitor of VV has never been clarified; the N-7-substituted acyclic nucleoside analog 2-amino-7-[(1,3-dihydroxy-2-propoxy)methyl]purine S2242 [52], which is inhibitory to VV, as well as various herpesviruses (HSV, VZV, CMV), and whose antiviral activity, while depending on phosphorylation by deoxycytidine kinase (dCK) [53], was never fully elucidated; and adenine arabinoside, vidarabine (ara-A), the first antiviral drug to be approved for systemic use (for the treatment of VZV infections) [54], which is quite effective against VV replication *in vitro* [31] (the inhibition of VV replication by ara-A may be explained by the inhibitory effect of ara-ATP on viral DNA synthesis and/or a direct inhibitory effect of ara-A on SAH hydrolase, akin to that of all other adenosine analogs mentioned above (see *supra*).

Of the compounds listed above, very few were further explored for their *in vivo* activity against VV infection, and, when this was done, uniformly the pox tail lesion model was used: hence, this model allowed demonstration of the *in vivo* efficacy of EICAR [17], S2242 [18] and 3-deazaneplanocin A [21]. When no further *in vivo* testing was done with most of the compounds showing sufficient potency against VV *in vitro*, this must be attributed to the paucity of material available for such *in vivo* testing.

**Figure 2:**
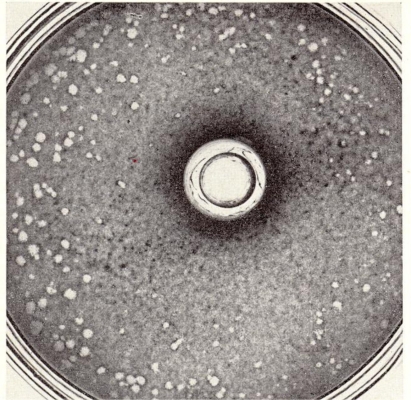
Large zone of inhibition without zone of toxicity. Cultures of chick fibroblast cells were seeded with approximately 500 plaque-forming units of vaccinia virus; the cylinder(s) were charged with 0.05 ml of solution of the substance tested (6-azauracil riboside)) (2.0 M); dishes of 4 cm diameter were employed. Figure taken from Rada, Blaškovič, Šorm and Škoda [57].

In part C of my Stories in Antiviral Drug Discovery [55] I wrote that the first description of the antiviral activity of 6-azauridine dated back to 1977, thereby referring to the paper published by Rada and Dragun in Ann. N.Y. Acad. Sci. [56]. This is not entirely correct. Rada, Blaškovič, Šorm and Škoda in 1960 (that is one year before Herrmann reported the plaque inhibition test for demonstrating the inhibitory effect of IDU on VV and HSV [25]) published a brief report in Experientia [57] on the inhibitory effect of 6-azauracil riboside on the multiplication of vaccinia virus (Figure 2). Rada *et al.* [57] also stated that 6-azauracil riboside had no direct inactivating effect on vaccinia virus and had no inhibitory effect on the multiplication of influenza virus, Newcastle disease virus (NDV) and Eastern equine encephalomyelitis virus when tested by the plaque method [57]. The mechanism of antiviral action of 6-azauridine was never fully elucidated. It has been assumed to interact, like pyrazofurin, at the level of the OMP decarboxylase [55].

## 5. The acyclic nucleoside phosphonates

In 1986, we (Antonin Holý and I, and our colleagues) described the broad-spectrum anti-DNA virals, including anti-VV, activity of (*S*)-9-(3-hydroxy-2-phosphonylmethoxypropyl)adenine [(*S*)-HPMPA] [58], and one year later, we extended these observations to various other phosphonylmethoxyalkyl derivatives of purines and pyrimidines, *i.e.,* (*S*)-1-(3-hydroxy-2-phosphonylmethoxypropyl)cytosine [(*S*)-HPMPC] [59] [(*S*)-HPMPC (cidofovir, Vistide®) would be licensed nine years later, in 1996, for the treatment of CMV retinitis in AIDS patients]. From the beginning, it was immediately clear that (*S*)-HPMPA and especially (*S*)-HPMPC (because it seemed to be less toxic in mice) had therapeutic potential as antiviral drugs [60]. The *in vivo* activity of (*S*)-HPMPA in the VV pox tail lesion model was already mentioned in the paper published in Antiviral Research in 1987 [59]. For (*S*)-HPMPC, we first focused on its efficacy in the therapy of experimental HSV infections in mice [61], before we described, in 1993, its efficacy in the treatment of VV infections in both normal, immunocompetent mice (based, again, on the VV pox tail lesion model) and immunodeficient (SCID) mice [62].

This was five years before orthopoxviruses, in particular variola, the etiological agent of smallpox, were, rather suddenly, felt as a bioterrorist public health threat [63,64]. This bioterrorist scenario prompted the search for an adequate therapy (or prophylaxis) to curb orthopoxvirus infections, and in the next few years several investigators (Bray *et al.*, Smee *et al*.) found (*S*)-HPMPC (in the mean time better known as cidofovir) to protect mice against lethal respiratory infections with cowpox virus [65,66] and vaccinia virus [67,68]. Aerosolized cidofovir was found to be efficacious against aerosolized cowpox virus infection in mice [69], and cidofovir also proved efficacious in the treatment of mice infected with an highly virulent strain of ectromelia (mousepox virus) encoding interleukin-4 [70].

**Figure 3:**
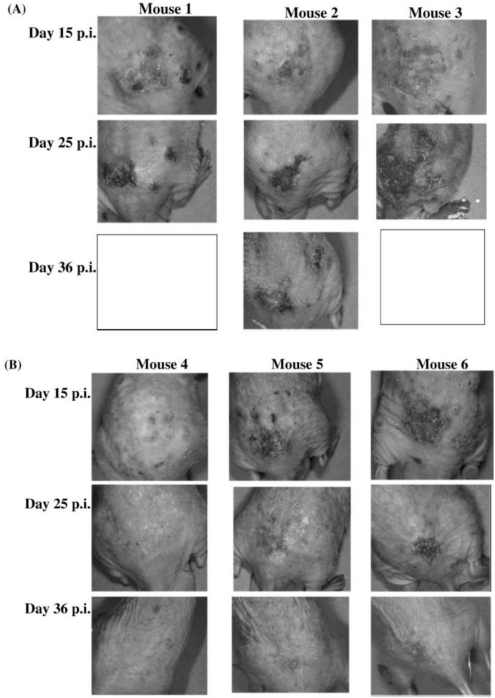
Effect of systemically administered cidofovir on disseminated vaccinia when therapy was initiated on day 15 p.i. **(A)** Progression of cutaneous vaccinia in three mock-treated animals. Mice 1 and 3 died on day 35 p.i.; mouse 2 died on day 36 p.i. **(B)** Effect of subcutaneous treatment with cidofovir (at 100 mg/kg/day), initiated on day 15 p.i., on disseminated cutaneous vaccinia lesions. Cidofovir was given for 21 days (over a period of 24 days). Figure taken from Neyts, Leyssen, Verbeken and De Clercq [72,73].

In a review on the potential of cidofovir in the treatment of poxvirus infections [71], I stated that cidofovir should be effective in the therapy and short-term prophylaxis of smallpox and related poxvirus infections in humans, as well as the treatment of the complications of vaccinia that may arise in immunocompromised patients inadvertently inoculated with the smallpox vaccine (vaccinia). With Johan Neyts and colleagues, I later demonstrated that cidofovir is indeed efficacious in the treatment of disseminated progressive vaccinia (in a mouse model); even when cidofovir treatment was initiated at the time disseminated vaccinia had developed, it caused lesions to heal and regress (Figure 3) [72,73].

## 6. Oral alkoxyalkyl prodrugs of acyclic nucleoside phosphonates

An additional advantage of cidofovir is that it is not only active against orthopoxviruses, but also parapoxviruses [such as orf (ecthyma contagiosum)] and molluscipoxviruses (such as molluscum contagiosum), which may both lead to serious infections in immunocompromised patients (in fact, anecdotal reports suggest that cidofovir is clinically efficacious against orf and molluscum contagiosum [71,74,75]). However, cidofovir, like all other acyclic nucleoside phosphonates [76], suffers from the disadvantage that it has poor oral bioavailability. This explains why alkoxyalkyl [*i.e.,* hexadecyloxypropyl (HDP) and octadecyloxyethyl (ODE)] prodrugs of cidofovir (HDP-CDV and ODE-CDV) were developed [77], intentionally as oral medicines for the prophylaxis and therapy of smallpox infection [78]. Esterification of cidofovir with alkoxyalkanols not only increases its oral bioavailability but also diminishes drug accumulation in the kidney [79], and thus reduces nephrotoxicity [77]. In molecular terms, HDP-CDV is rapidly taken up in the cells thanks to a rapid association with cellular membrane phospholipids [80]. This results in enhanced inhibition of orthopoxvirus (*i.e.,* vaccinia virus and cowpox virus) replication *in vitro* [81,82]. Increased *in vivo* activity of HDP-CDV and ODE-CDV, over that of CDV, has also been demonstrated in mice infected with vaccinia virus (VV) or cowpox virus (CV) [83], or ectromelia (mousepox) virus [84].

Following the same reasoning as for cidofovir, alkoxyalkyl (*i.e.,* HDP and ODE) derivatives have also been prepared from (*S*)-HPMPA, the prototype of the acyclic nucleoside phosphonates [58]. When compared with the alkoxyalkyl esters of cidofovir, the corresponding alkoxyalkyl esters of (*S*)-HPMPA were equally active against CMV but were 15- to 20-fold more active against VV and CV *in vitro* [85]. *In vivo*, oral treatment with HDP-(*S*)-HPMPA or ODE-(*S*)-HPMPA were highly effective against lethal VV or CV infections in mice [86]. Thus, alkoxyalkyl prodrugs of both cidofovir and (*S*)-HPMPA can be considered excellent candidates for the oral treatment (and prophylaxis) of smallpox in humans, should this prove necessary in the event of a bioterrorist release of the variola virus (HDP-CDV is also referred to as CMX001 [78]).

## 7. ST-246, an orally bioavailable antipoxvirus compound, that inhibits extracellular virus formation

Following a totally new strategy (different from the nucleoside or nucleotide analogs), Dennis Hruby and his colleagues at SIGA Technologies in Corvallis (Oregon) came up with a new class of orthopoxvirus (*i.e.,* vaccinia virus) antiviral drugs (*i.e.,* TTP-6171) that block viral maturation [87]. These drugs block viral maturation through an interaction with the VV I7L gene product, a cysteine proteinase responsible for cleavage of the three major core protein precursors [88] and, hence, the production of infectious virions [89]. The reported compound, TTP-6171, was not further pursued as a potentially antiviral drug, in favor of a newly discovered compound, ST-246, that appeared to inhibit extracellular orthopoxvirus formation (and to protect mice from a lethal orthopoxvirus challenge) [90], apparently as the result of an action targeted at the F13L protein (phospholipase) which had previously [91] been recognized as a major membrane component of extracellular vaccinia virus.

ST-246, or 4-trifluoromethyl-*N*-(3,3a,4,4a,5,5a,6,6a-octahydro-1,3-dioxo-4,6-etheno-cycloprop-(f)isoindol-2-(1*H*-yl)benzamide [92] was shown to have activity against a number of orthopoxviruses, including vaccinia, monkeypox, camelpox, cowpox, ectromelia (mousepox) and variola virus [90]. ST-246 has proven effective against vaccinia, cowpox, and camelpox viruses in human embryonic lung and primary human keratinocyte cell monolayers, as well as three-dimensional organotypic raft cultures [93]. *In vivo* efficacy of ST-246 has been demonstrated in mice infected with vaccinia virus [90] or ectromelia virus (even if treatment was delayed until 72 hours after virus infection [94]), and in ground squirrels infected with monkeypox virus [95]. ST-246 is also effective against aerosolized rabbit pox in rabbits [96], and acts synergistically with CMX001 (HDP-CDV) in mice infected with cowpox virus [97], which opens new avenues for the treatment of orthopoxvirus infections.

Most important is to know whether ST-246 would be effective against smallpox (bioterrorist attack) or the complications of smallpox vaccination (such as eczema vaccinatum). A recent case of severe eczema vaccinatum in a household contact (28-month-old son) of a smallpox vaccinee illustrates the risks for such adverse events [98]. The boy eventually recovered after treatment with vaccinia immune globulin, cidofovir, and ST-246 [98]. What exactly saved the boy’s life - the antibody, cidofovir, ST-246, or the extraordinary medical care, or combinations of some or all of the above - would never be known.

Recently, ST-246 has been found to protect non-human primates from smallpox virus or monkeypox virus [99]. ST-246 has, in the mean time, been renamed tecovirimat [100] and its target protein, F13L, is also known as p37. It is further developed by SIGA Technologies for the treatment of variola virus infection (smallpox) [100].

## 8. Cellular kinases as targets for anti-orthopoxvirus therapy

Among the various agents that could be considered potentially useful in the therapy of (ortho)poxvirus infections [101], also rank a few compounds that are targeted at cellular (tyrosine) kinases, and thus could inhibit poxvirus infections through inhibition of cellular signal transduction, *i.e.,* CI-1033 and related 4-anilinoquinazolines [102] and STI-571 (imatinib, better known as Gleevec or Glivec) [103]. Gleevec has been licensed for clinical use as an anticancer agent, to treat chronic myeloid leukemia. That it would also have the ability to inhibit poxvirus infections (by blocking the egress of these viruses from infected cells) [103] is an unanticipated attribute [104]. Thus, Gleevec casts “a pox on poxviruses” [104], but although the findings of Reeves *et al.* [103] date from five years back, the potential utility of Gleevec, or tyrosine kinase inhibitors in general, in the treatment of poxvirus infections like smallpox, monkeypox, or the complications associated with vaccinia virus vaccination, still remains to be substantiated.

## 9. Camelpox virus, the orthopoxvirus most closely related to variola virus

Of all the (ortho)poxviruses, camelpox virus, the etiologic agent of camelpox occurring in Old World camels (*Camelus dromedarius* and *Camelus bactrianus*), is the orthopoxvirus most closely related to variola virus, the cause of smallpox [105]. Studies with camelpox virus (which is not supposed to be pathogenic for humans) have allowed confirmation of the anti-poxvirus activity of the “older” acyclic nucleoside phosphonates such as (*S*)-HPMPA and (*S*)-HPMPC [106], but also to extend the anti-camelpox virus activity to the “newer” acyclic nucleoside phosphonates such as (*R*)-{2,4-diamino-3-hydroxy-6-[2-(phosphonomethoxy)propopy]}pyrimidine [(*R*)-HPMPO-DAPy] and 1-(*S*)-[3-hydroxy-2-(phosphonomethoxy)propyl]-5-azacytosine (HPMP-5-azaC) [107]. As already mentioned above [93], the anti-(ortho)poxvirus activity of ST-246 also extends to camelpox virus, although camelpox, vaccinia, and cowpox show different levels of sensitivity to ST-246, probably because of dissimilarities between their ways of propagation [108].

## 10. Epilogue: antiviral treatment more effective than smallpox vaccination

**Figure 4:**
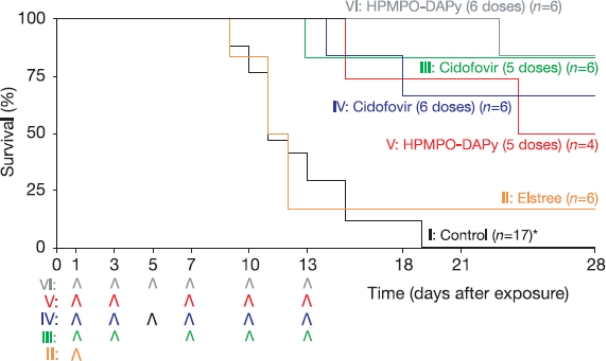
Kaplan-Meier plot showing the effect of different post-exposure treatments on the survival of monkeys. Survival expressed as percentages after intratracheal MPXV infection. After virus exposure, macaques were sham-treated (group I), vaccinated with smallpox vaccine Elstree-RIVM (group II), treated with 5 doses of cidofovir (group III), with six doses of cidofovir (group IV), with five doses of HPMPO-DAPy (group V) or with six doses of HPMPO-DAPy (group VI). Arrowheads indicate times of vaccination and antiviral treatment. *The control group is supplemented with 11 animals from previous experiments. Figure taken from Stittelaar *et al*. [109].

Although the preventive use of smallpox vaccination is well documented, little is known about its efficacy when used after exposure to variola virus. A few years ago, we (Albert Osterhaus and I, and our colleagues) wanted to compare the effectiveness of post-exposure smallpox vaccination and antiviral treatment with either cidofovir [(*S*)-HPMPC] or the related acyclic nucleoside phosphonate analog (*R*)-HPMPO-DAPy, at 24 hours after lethal intratracheal infection of cynomolgus monkeys (*Macaca fascicularis*) with monkeypox virus. This study was actually planned after I had met Koert Stittelaar at a meeting in London (“Twenty-five years on: smallpox revisited”, Retroscreen Virology and Institute of Cell and Molecular Sciences Barts and the London Queen Mary’s School of Medicine & Dentistry, The Great Hall, St. Bartholomew’s Hospital, London, Great Britain, 31 October 2003). This study, published in 2006 in Nature [109] clearly revealed that initiation of antiviral treatment with either cidofovir or (*R*)-HPMPO-DAPy at 24 hours after monkeypox virus infection resulted in significantly reduced mortality (Figure 4). In contrast, when monkeys were vaccinated at 24 hours after virus infection, using a standard human dose of a currently recommended smallpox vaccine (Elstree), no significant reduction in mortality was observed [109]. From this study [109], we concluded “that adequate preparedness for a biological threat involving smallpox should include the possibility of treating exposed individuals with antiviral compounds such as cidofovir or other selective anti-poxvirus drugs”.

## Appendix



## Figures and Tables

**Table 1:** Effect of time of a single polyacrylic acid (PAA) dose on the formation of vaccinia virus tail lesions. Data taken from De Clercq and De Somer [11].

PAA treatment^a^ before vaccinia virus infection^b^	No. of lesions		Probability (comparison with control group)
	Per individual mouse	Average	
Control	9, 3, 8, 1, 6, 2, 5, 11, 4, 0, 3, 2, 0, 11, 15, 10, 30, 4	6.89	
4 weeks before	0, 1, 0, 5, 1, 0, 2, 0, 3, 1, 0, 2, 1, 3, 0, 11, 4, 3, 7, 1, 0	2.14	0.005 < *P* < 0.01
3 weeks before	0, 0, 1, 3, 5, 3, 0, 0, 0, 9, 3, 1, 0, 0, 1	1.73	0.01 < *P* < 0.02
2 weeks before	3, 1, 2, 1, 2, 1, 6, 1, 0, 0, 0, 1, 2, 1	1.50	*P* ∼ 0.01
1 week before	0, 1, 1, 0, 0, 3, 2, 1, 1, 0, 2, 2, 1, 0, 1	1.00	0.001 < *P* < 0.002

a PAA (0.25 mg) injected intraperitoneally.

b Vaccinia virus injected intravenously.
