# Supplementary material for: Historical Perspectives in the Development of Antiviral Agents Against Poxviruses
Source: Viruses. 2010 Jun 14;2(6):1322–39. doi: 10.3390/v2061322 (PMC3185982; doi:10.3390/v2061322)
Supplement: Supplementary file 1 [file viruses-02-01322-s001.pdf]

Appendix

Structural formulae of compounds mentioned in the manuscript

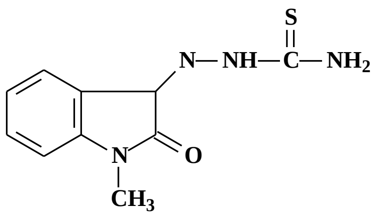

Methisazone
N-methylisatin 3-thiosemicarbazone
Marboran®

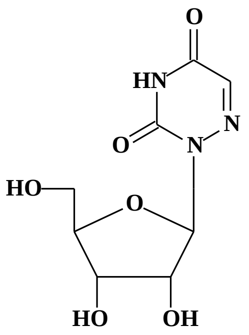

6-Azauridine
6-Azauracil riboside

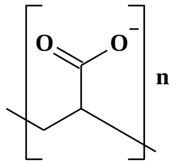

PAA
Poly(acrylic acid)

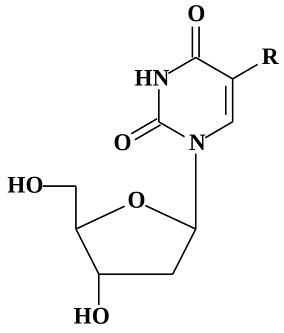

5-Substituted 2'-deoxyuridines
R = I : 5-iodo-2'-deoxyuridine, idoxuridine, IDU
= CF_3 : 5-trifluoromethyl-2'-deoxyuridine, trifluridine, TFT

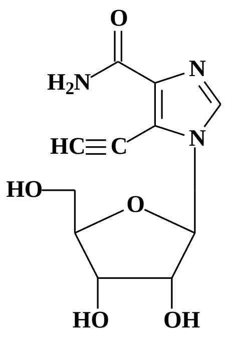

EICAR

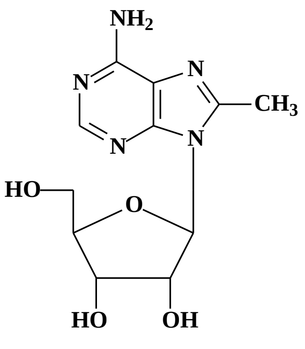

8-Methyladenosine

Appendix *Cont.*

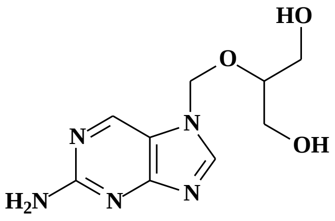

S2242
2-Amino-7-[(1,3-dihydroxy-2-propoxy)methyl]purine

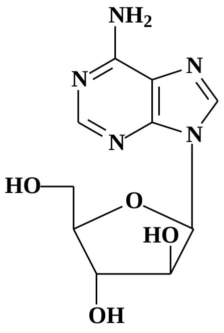

Adenine arabinoside
Ara-A
Vidarabine
Vira-A®

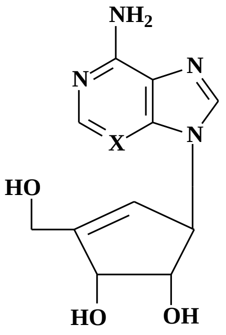

Neplanocin A (X = N)
3-Deazaneplanocin A (X = CH)

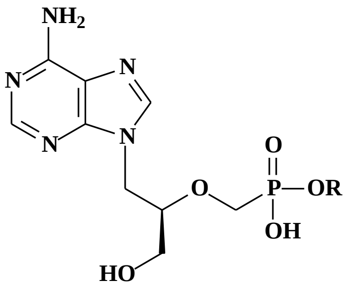

R = H: (*S*)-HPMPA
= $(CH_2)_3O(CH_2)_{15}CH_3$: HDP-(*S*)-HPMPA

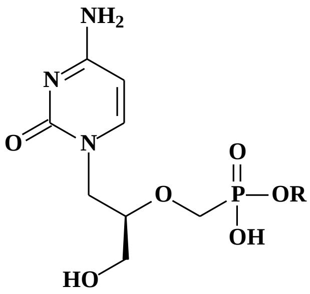

R = H: (*S*)-HPMPC (Cidofovir)
= $(CH_2)_3O(CH_2)_{15}CH_3$: HDP-Cidofovir
= $(CH_2)_2O(CH_2)_{17}CH_3$: ODE-Cidofovir

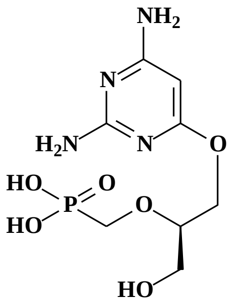

(*R*)-HPMPO-DAPy

Appendix *Cont.*

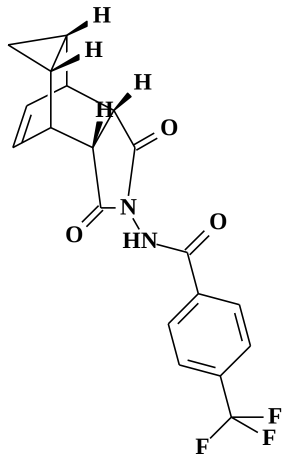

ST-246
Tecovirimat

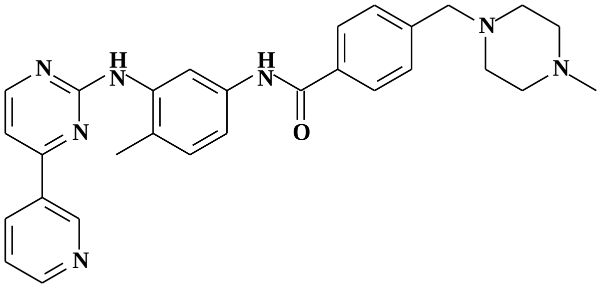

Imatinib
STI-571
Gleevec®

© 2010 by the authors; licensee MDPI, Basel, Switzerland. This article is an Open Access article distributed under the terms and conditions of the Creative Commons Attribution license (http://creativecommons.org/licenses/by/3.0/).
